# A new species of *Caecilia* (Gymnophiona, Caeciliidae) from the Magdalena valley region of Colombia

**DOI:** 10.3897/zookeys.884.35776

**Published:** 2019-10-30

**Authors:** Andrés R. Acosta-Galvis, Mauricio Torres, Paola Pulido-Santacruz

**Affiliations:** 1 Colecciones Biológicas, Subdirección de Investigaciones, Instituto de Investigación de Recursos Biológicos Alexander von Humboldt, Carrera 8 No 15–08, Claustro de San Agustín, Villa de Leyva, Boyacá, Colombia; 2 Subdirección de Investigaciones, Instituto de Investigación de Recursos Biológicos Alexander von Humboldt, Calle 28a No. 15–09, Bogotá, Colombia; 3 Genética de la Conservación, Instituto de Investigación de Recursos Biológicos Alexander von Humboldt, Calle 28a No. 15–09, Bogotá, Colombia

**Keywords:** Amphibia, *Caecilia degenerata*, *
Epicrionops
*, *
Microcaecilia
*, paraphyly, phylogeny, Siphonopidae, South America, taxonomy, tropical humid forest

## Abstract

A new species of the genus *Caecilia* (Caeciliidae) from the western foothills of the Serranía de los Yariguíes in Colombia is described. *Caecilia pulchraserrana***sp. nov.** is similar to *C. degenerata* and *C. corpulenta* but differs from these species in having fewer primary annular grooves and a shorter body length. With this new species, the currently recognized species in the genus are increased to 35. Mitochondrial DNA sequences, including newly sequenced terminals representing two additional, previously unanalyzed species, corroborate the phylogenetic position of the new species within *Caecilia* and the monophyly of the genus. This analysis also included newly sequenced terminals of Epicrionops aff. parkeri (Rhinatrematidae) and trans-Andean *Microcaecilia nicefori* (Siphonopidae). Evidence was found for the non-monophyly of the family Siphonopidae and the siphonopid genera *Microcaecilia* and *Siphonops*. The implications of these results for caecilian systematics are discussed and the status of the trans-Andean populations of *Caecilia degenerata* is commented upon.

## Introduction

The Neotropical caecilian amphibian genus *Caecilia* Linnaeus, 1758 (Gymnophiona: Caeciliidae) currently comprises 34 nominal species (Wilkinson et al. 2011; Frost 2018; Maciel and Hoogmoed 2018), 18 of which occur in Colombia, with eight being endemic to this country. Seven species occur in the Magdalena valley region of Colombia (Dunn 1942; Lynch 1999) and external morphology segregates them into two groups. A first group comprises four species that lack secondary annular grooves: *C. caribea* Dunn, 1942, endemic to Colombia, from the eastern slope of the Cordillera Central, Caldas Department, between 10–1700 m above sea level (a.s.l); *C. corpulenta* Taylor, 1968, from the type locality in Peru, with a Colombian record from the Andean forests on the 1750 m a.s.l., Santander Department; *C. subdermalis* Taylor, 1968, from northern Ecuador and eastern slopes of the Cordillera Central, Huila and Caldas Departments in Colombia, between 850–2320 m a.s.l.; and *C. degenerata* Dunn, 1942, endemic to Colombia, from both flanks of the Cordillera Oriental, between 800–2100 m a.s.l., Boyacá, Cundinamarca, and Santander Departments (Dunn 1942; Taylor 1968; Ruiz-Carranza et al. 1996; Lynch 1999; Acosta-Galvis 2000; Rivera-Correa 2006; Castro-Herrera et al. 2007; Frost 2018; Appendix 1).

A second group includes three species that have secondary annular grooves: *C. guntheri* Dunn, 1942, with a wide distribution from northern Ecuador to Colombia, where the records are discontinuous and include the sub-Andean forests of the Cordillera Occidental and the region of Muzo at Quípama Municipality, Boyacá Department, western slope of the Cordillera Oriental, 1000 m a.s.l.; *C. subnigricans* Dunn, 1942, from northern Venezuela and lowlands of the Caribbean and Magdalena Valley regions of Colombia, with a record from Mariquita Municipality, Tolima Department; and *C. thompsoni* Boulenger, 1902b, endemic to the middle Magdalena valley in Colombia, 240–1571 m a.s.l. (Dunn 1942; Taylor 1968; Ruiz-Carranza et al. 1996; Lynch 1999; Acosta-Galvis 2000; [Bibr B5][Bibr B3]; Lynch and Romero 2012; Mueses-Cisneros and Moreno-Quintero 2012; Paternina-H et al. 2013; Acevedo-Rincón et al. 2014; Angarita-M et al. 2015; Restrepo et al. 2017; Frost 2018; Appendix 1).

**Figure 1.**
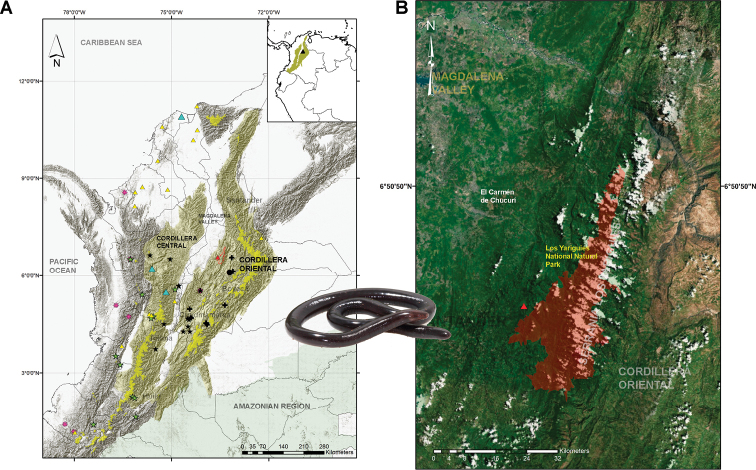
**A** Map of Colombia showing the known localities of the species of *Caecilia* that occur in the Magdalena valley region. Key: *C. caribea* (blue triangle), *C. corpulenta* (black dot), *C. degenerata* (black cross), *C. guntheri* (violet asterisk), *C. subnigricans* (yellow triangle), *C. subdermalis* (green star), *C. thompsoni* (black star), *Caecilia pulchraserrana* sp. nov. (red triangle) **B** Type locality of *Caecilia pulchraserrana* sp. nov. (red triangle) at Serranía de los Yariguíes, Santander Department, Colombia.

During a recent herpetological survey in wet tropical forests of the Serranía de los Yariguíes, in the Department of Santander, Colombia (Fig. 1), we collected several specimens of a small *Caecilia* that lack secondary annular grooves and dermal scale pockets, suggesting that they correspond to either *C. degenerata* or *C. corpulenta*. However, a low number of primary annular grooves and a combination of morphometric characters indicate instead that these specimens belong to a new species, which we describe herein. To test the generic placement of the new species and to explore the relationships of other Neotropical caecilians, we perform a phylogenetic analysis of DNA sequences. We discuss the implications of our results for caecilian systematics and comment on the status of the trans-Andean populations of *C. degenerata*.

## Materials and methods

### Fieldwork and reference collections

The new species was collected during fieldwork carried out in the Serranía de los Yariguíes, vereda La Belleza, municipality of El Carmen de Chucurí, Santander Department, Colombia (06°34'N, 73°34'W, 731–789 m a.s.l.; Fig. 1), from 17 February to 1 May 2018, during the dry season. Specimens were found in two separate humid spots near the Río Cascajales, which drains Tropical moist broadleaf forests, within the ecoregion of the Magdalena valley montane forests, in the foothills of the Cordillera Oriental, Colombia (Dinerstein et al. 1995; Olson and Dinerstein 2002).

Previous fieldwork conducted between 1998–1999 by John Lynch in collaboration with the first author, successfully allowed the detection of microhabitats and several specimens of *Microcaecilia nicefori* (Lynch 1999); subsequently, between 2000 to date, fieldwork with caecilians such as *Oscaecilia polyzona* (Lynch and Acosta 2004), *Caecilia* sp., *C. thompsoni*, and *C. isthmica* (unpublished data) allowed successful detection of microhabitats and multiple specimens.

The collecting technique, which was used to obtain specimens of the new species, consists of first asking local people about the locations where they have spotted caecilians using the common names of “blind snakes”, or “captain worms” (“lombrices capitanas”), or “motolas” (this common name is specific for the Department of Santander). Subsequently, the reported sites are visited and inspected to select sites under the shade of vegetation, and where the soil is not compact and very humid (usually associated with water springs that form a mosaic of marshy and dry areas). Collecting efforts are focused in the selected damp microhabitats, digging with a hoe to a depth of approximately 20 cm (approximate sampling effort of 2-person-hour to collect five specimens). Coordinates and elevations were obtained with a Garmin GPSMAP 64SC (map datum WGS 84). Collected specimens were euthanized using 20% benzocaine (Chen and Combs 1999), fixed in 10% formalin, and preserved in 70% ethanol. Tissue samples from two individuals were obtained immediately after euthanasia and preserved in 96% ethanol. Specimens were deposited at the Biological Collections of the Instituto de Investigación de Recursos Biológicos Alexander von Humboldt, Villa de Leyva, Boyacá, Colombia (**IAvH-Am** and **IAvH-CT**) and the Amphibian Collection of the Universidad Industrial de Santander, Bucaramanga, Santander, Colombia (**UIS-MHN-A**).

### Phylogenetic analysis

To test the generic assignment of the new species and to explore the relationships of other endemic caecilians from Colombia, available mitochondrial DNA sequences of the genes 16S and CO1 from members of all Neotropical caecilian families (Caeciliidae, Typhlonectidae, Siphonopidae, Dermophiidae, and Rhinatrematidae) were analyzed (Table 1). The analysis included a fragment of COI (ca. 651 bp) and a fragment of 16S (ca. 510 bp). Sequences for most terminals were obtained from GenBank (Table 1). We added new sequences for eight Colombian terminals representing the new species, *Caecilia thompsoni*, *C. isthmica*, *Typhlonectes natans*, Epicrionops aff. parkeri, and *Microcaecilia nicefori* (Appendix 1). The cryptobranchid *Cryptobranchus alleganiensis* was used to root the tree. Laboratory protocols and primers are those of Palumbi (1996), Ivanova et al. (2006), and Carr et al.(2011). Bidirectional PCR products were used for Sanger sequencing at the Instituto de Genética of the Universidad Nacional de Colombia. Resulting sequences were visualized, assembled, checked for stop codons (COI), and edited in Geneious Pro v 10.2.3 (Kearse et al. 2012). All sequences were deposited in the Barcode of Life Data System (BOLD; Ratnasingham and Hebert 2007) and GenBank (Table 1). Sequences of each gene were aligned independently using the MAFFT plugin *v* 7.388 within Geneious, considering the secondary structure of RNA in 16S and implementing the G-INS-I algorithm. Subsequently, sequences of both genes were concatenated in a single dataset using Geneious, which was used to construct a Maximum Likelihood phylogeny using IQ-TREE (Nguyen et al. 2015), performing a partitioned analysis based on four partitions (16S, COI first codon position, COI second codon position, COI third codon position) using the partition finder algorithm (-m option TESTMERGE; Lanfear et al. 2012) in IQ-TREE and best fitting models for each partition selected by the same program (Chernomor et al. 2016; Kalyaanamoorthy et al. 2017). Each partition was allowed to have its own set of branch lengths (-sp option). Branch support analysis was performed with 1000 ultrafast bootstrap replicates (Hoang and Chernomor 2017).

**Table 1.** List of species examined and GenBank or Barcode of life Data Systems (**BOLD**) accession numbers for each gene analyzed in this study. See Appendix 1 for locality details.

Species	Family	Tissue code	16S GenBank; BOLD number	CO1 GenBank; BOLD number	Source
*Caecilia gracilis*	Caeciliidae		KX757086	NC_023508	Maciel et al. 2017, San Mauro et al. 2014
*Caecilia isthmica*	Caeciliidae	IAvH-CT-22982	MN555719; SABIO393-19	MN555727; SABIO393-19	This study
*Caecilia pulchraserrana* sp. nov	Caeciliidae	IAvH-CT-227334	MN555715; SABIO005-18	MN555723; SABIO005-18	This study
	Caeciliidae	IAvH-CT-22733	MN555718; SABIO002-18	MN555726; SABIO002-18	This study
*Caecilia tentaculata*	Caeciliidae		NC_023507	NC_023507	San Mauro et al. 2014
*Caecilia thompsoni*	Caeciliidae	IAvH-CT-22986	MN555717; SABIO392-19	MN555725; SABIO392-19	This study
*Caecilia volcani*	Caeciliidae		FJ784371	NC_020137	Crawford et al. 2010, Zhang and Wake 2009
*Oscaecilia ochrocephala*	Caeciliidae		GQ244474	GQ244474	Zhang and Wake 2009
*Dermophis mexicanus*	Dermophiidae		–	NC_020138	Zhang and Wake 2009
Epicrionops cf. marmoratus	Rhinatrematidae		KF540151	KF540151	San Mauro et al. 2014
*Rhinatrema nigrum*	Rhinatrematidae		GQ244468	GQ244468	Zhang and Wake 2009
Epicrionops aff. parkeri	Rhinatrematidae	IAvH-CT-21477	MN555716; CBIHA031-17	MN555724; CBIHA031-17	This study
*Microcaecilia dermatophaga*	Siphonopidae		NC_023514	NC_023514	San Mauro et al. 2014
*Microcaecilia* sp.	Siphonopidae		GQ244473	GQ244473	Zhang and Wake 2009
*Microcaecilia unicolor*	Siphonopidae		NC_023515	NC_023515	San Mauro et al. 2014
*Microcaecilia nicefori*	Siphonopidae	IAvH-CT-22985	MN555722; CAECI002-19	MN555729; CAECI002-19	This study
*Siphonops annulatus*	Siphonopidae		KU495581	KU495581	Lyra et al. 2017
*Siphonops hardyii*	Siphonopidae		KU495582	KU494789	Lyra et al. 2017
*Siphonops insulanus*	Siphonopidae		KU495583	KU494790	Lyra et al. 2017
*Siphonops paulensis*	Siphonopidae		KU495584	KU494791	Lyra et al. 2017
*Potomotyphlus kaupii*	Typhlonectidae		NC_023516	NC_023516	San Mauro et al. 2014
*Typhlonectes compressicauda*	Typhlonectidae		KU495605	KU494812	Lyra et al. 2017.
*Typhlonectes natans*	Typhlonectidae		AF154051	AF154051	Zardoya and Meyer 2000.
	Typhlonectidae	IAvH-CT-22983	MN555720; SABIO394-19	MN555728; SABIO394-19	This study
	Typhlonectidae	IAvH-CT-22984	MN555721; CAECI001-19	–	This study

### Morphology

Criteria and terminology for morphological descriptions, diagnostic characters, and data for other species of *Caecilia* follow Lynch (1999), Gower and Wilkinson (2002), Maciel et al. (2009), Maciel and Hoogmoed (2011), Kamei et al. (2009), Wilkinson and Kok (2010), Donnelly and Wake (2013), and Wilkinson et al. (2009, 2013, 2015). For comparative purposes, specimens of *C. guntheri*, *C. isthmica*, *C. thompsoni*, and *C. subdermalis* were examined (Appendix 1). Morphological observations were made using a stereoscope Nikon optical device SMZ-1B, with High Intensity Illuminator NI-150 Nikon and App Scope 3xSRA41. Measurements were taken using a Mitutoyo precision digital caliper to ± 0.1 mm; and using the following abbreviations for anatomical features and ratios of measurements:

**ADD** anal disc diameter;

**AM** anteromedial limit of the mouth on the upper jaw;

**BH** body height at midbody;

**C1** first collar length;

**C2** second collar length;

**CM** corner of the mouth;

**CMB** circumference at midbody;

**D** diameter at midbody;

**ED** eye diameter;

**END** distance between eye and naris;

**HH** head height at level with CM;

**HL** head length;

**HW** head width at CM;

**HWNG1** head width at NG1;

**IND** distance between nares;

**IOD** interorbital distance;

**TL** total length;

**TL/D**TL divided by diameter at midbody (ratio of length/diameter);

**LPOD** distance between eye and lip;

**ND** naris diameter;

**NG1** first nuchal groove;

**NG2** second nuchal groove;

**NG3** third nuchal groove;

**PA** primary annulus;

**PAG** primary annular groove;

**PM** premaxillary-maxillary tooth;

**ST** snout tip;

**STD** distance between snout tip and anterior margin of mouth;

**STND** distance between ST and naris;

**STLPD** distance between ST and lip;

**STOD** distance between ST and eye;

**TA** tentacular aperture;

**INTA** distance between TAs;

**TAOD** distance between TA and eye;

**TALPD** distance between TA and lip;

**TANRD** distance between TA and naris;

**TASTD** distance between TA and ST;

**VP** vomeropalatine tooth;

**WC2** width at second collar;

**WCH** width of choanae;

**WBV** width of body at vent level;

**WMB** width at midbody;

**TL/HL**TL divided by HL;

**TL/WMB**TL divided by WMB;

**TL/HW**TL divided by HW;

**HL/HW**HL divided by HW.

Dermal scale pockets and subdermal scales were searched using the criteria proposed by Wilkinson et al. (2013) and sex and maturity were determined by examination of gonads. Live specimens were photographed with a digital camera model Canon EOS 70D and preserved specimens with a digital camera Canon EOS 5D Mark II.

## Results

### Phylogenetic analysis

**Figure 2.**
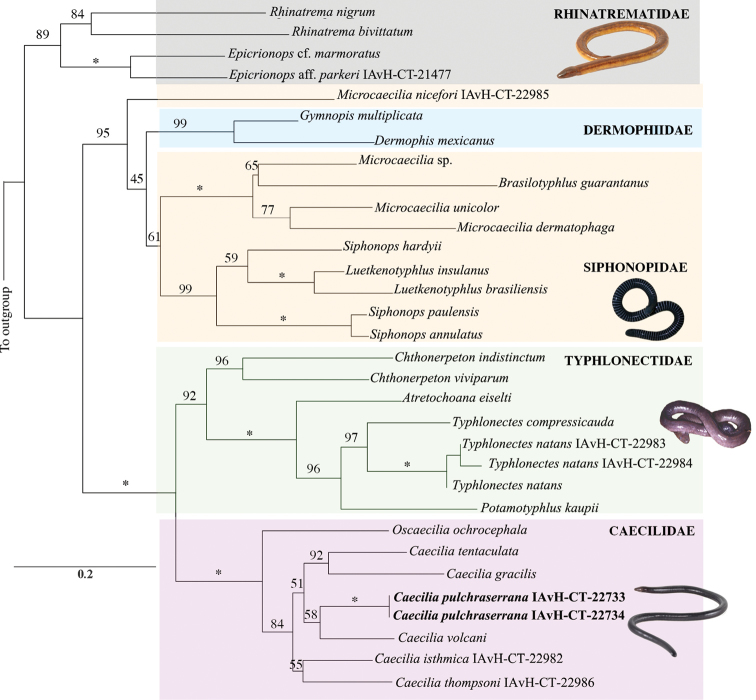
Maximum Likelihood tree inferred from the analysis of a concatenated dataset comprising partial sequences of two mitochondrial genes. Numbers above branches indicate bootstrap support values (percent) (* = 100% bootstrap). Scale bar indicates nucleotide substitutions per site. The phylogenetic position of *Caecilia pulchraserrana* sp. nov. is shown in bold.

The final concatenated molecular dataset consisted of a matrix of 1273 bp, 567 sites were parsimony-informative, 111 were singletons, and 595 were constant sites. The best fitting substitution model for both CO1 and 16S was TIM2+F+I+G4 after testing the large selection of models in IQ-TREE. The ML tree is shown in Fig. 2 (LnL: –15725.921). Our phylogenetic analysis recovered the new species nested within a moderately well-supported (84%) monophyletic *Caecilia*, in a maximally supported monophyletic Caeciliidae. The new species appears most closely related, of the sampled species, to *C. volcani* but support for this relationship is not strong (58%). *Rhinatrema nigrum* and *R. bivittatum* were recovered as monophyletic with the sister group *Epicrionops*. *Siphonops* was inferred to be paraphyletic with respect to *Luetkenotyphlus* (Siphonopidae), and *Microcaecilia nicefori* was recovered as the sister group of Dermophidae + Siphonophidae, the latter including the remaining *Microcaecilia* (with *Brasilotyphlus guarantanus* nested within it) and the paraphyletic *Siphonops*.

### Description of new species

**Generic assignment.** The new species is assignable to the genus *Caecilia* because its eyes are not covered by bone and it has tentacles below the nostrils (Type D sensu Lynch, 1999, Fig. 3 D–E). In addition, the new species is nested within the *Caecilia* clade (Fig. 2) in our Maximum Likelihood phylogenetic analysis.

**Figure 3.**
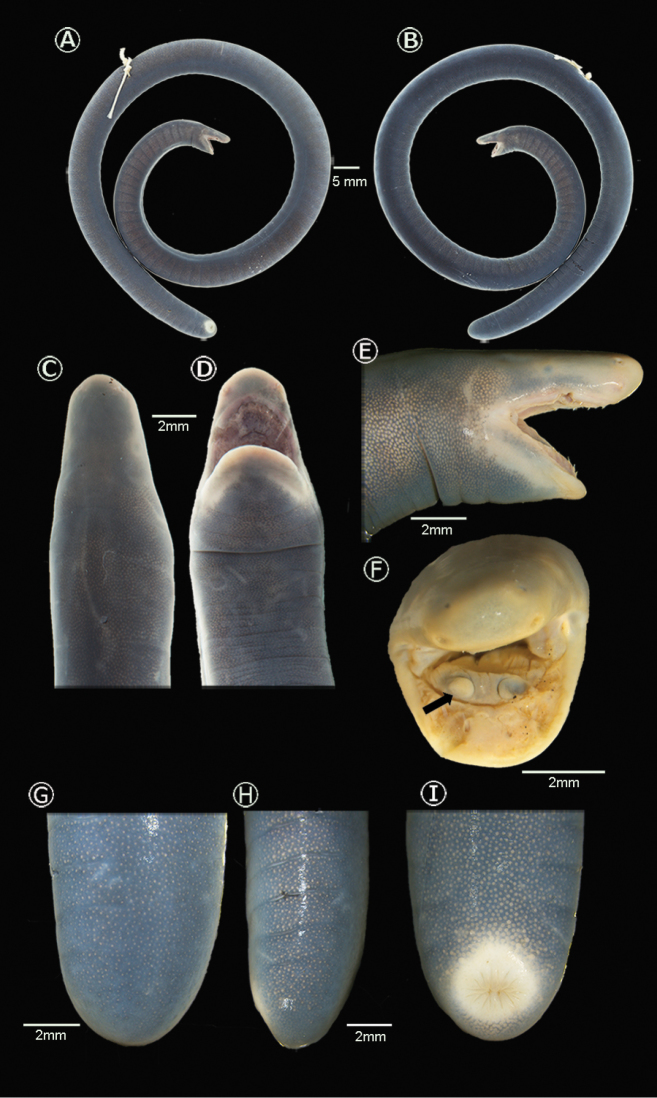
Holotype of *Caecilia pulchraserrana* sp. nov. Adult female, IAvH-Am-1548. **A, B** Lateral views of body **C** dorsal **D** ventral **E** lateral views of head **F** Frontal view of cephalic region, the arrow indicates the narial plug **G** dorsal and **H** lateral views of caudal region **I** ventral view of vent.

#### Caecilia pulchraserrana sp. nov.

Taxon classificationAnimaliaGymnophionaCaeciliidae

BD849A1C-D33F-5BA0-BB88-96D143F70831

http://zoobank.org/03F213A5-2148-4255-91BB-37719EF0E7B7

**Figure 4.**
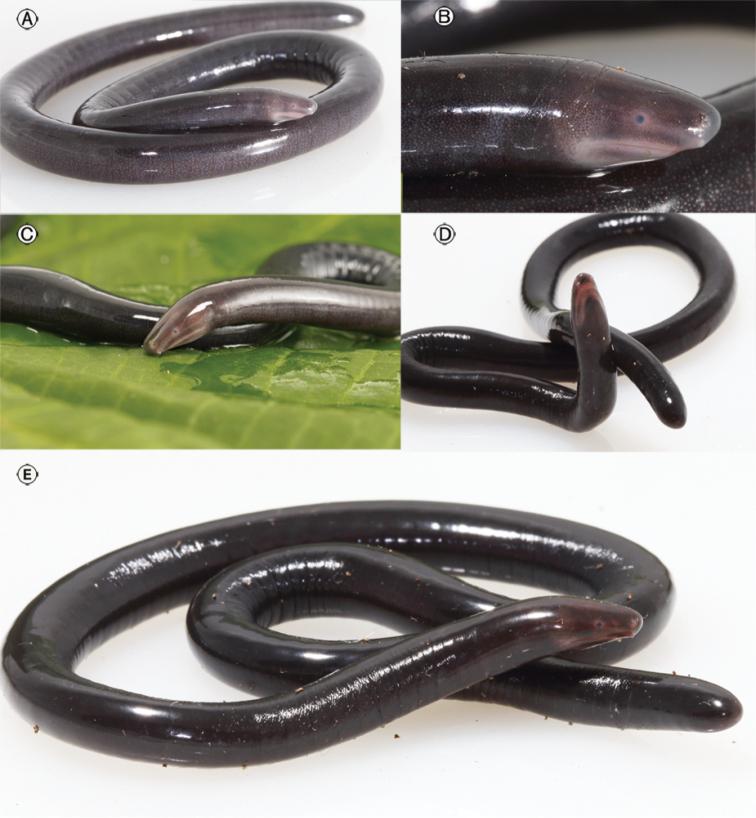
*Caecilia pulchraserrana* sp. nov. in life. **A** Adult female, paratype, IAvH-Am-15488, TL= 232 mm **B** adult female, paratype, IAvH-Am-15488, TL= 232 mm **C** adult female, holotype, IAvH-Am-15487, TL= 206 mm **D–E** adult female. paratype, UIS-MHN-A-6575, TL= 195 mm.

**Figure 5.**
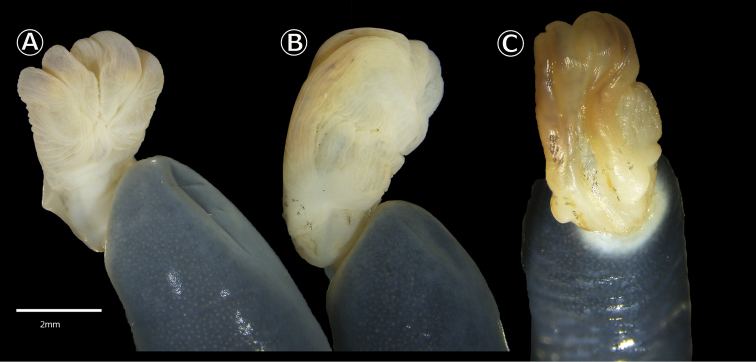
Phallus (everted cloaca) of adult males *Caecilia pulchraserrana* sp. nov. **A** Ventro-lateral view (IAvH-Am-15489) **B** Ventro-lateral view of vent and **C** dorsal surface of the phallus (IAvH-Am-15490).

Figs 3 , 4 , 5 ; Tables 2 , 3 , 4

##### Holotype.

IAvH-Am-15487 (field number ARA 7872; Figs 3, 4C), an adult female collected 25 February 2018 by A. R. Acosta-Galvis, Miguel Torres, and Daniela García.

##### Type Locality.

(Fig. 1) Colombia, Santander Department, El Carmen de Chucurí Municipality, vereda La Belleza, Cascajales River, 06°34'8.9"N, 73°34'20.2"W, 789 m a.s.l.

##### Paratypes.

Four specimens (Fig. 4), IAvH-Am-15488 (field number ARA 7871) and UIS-MHN-A-6575 (field number ARA 7689), adult females, collected with holotype, and IAvH-Am-15489–90 (field numbers ARA 7690–1, respectively), adult males (exhibiting phallus, Fig. 5 A–C), 06°34'41.1"N, 73°34'28.9"W, 731 m a.s.l., collected 19 February 2018 by A. R. Acosta-Galvis and Miguel Torres.

##### Referred specimens.

UIS-MHN-A-6576–7 (field numbers ARA 7692–3, respectively), juveniles, 06°34'41.1"N, 73°34'28.9"W, 731 m a.s.l., collected 19 February 2018 by A. R. Acosta-Galvis and Miguel Torres. Tissues for molecular analysis (IAvH-CT-22733–4) were extracted from these specimens.

##### Diagnosis.

*Caecilia pulchraserrana* sp. nov. differs from its congeners by the combination of having 100–104 dorsally incomplete primary annular grooves, a small size (195–232 mm), lips and ventral margin of upper jaw with a pink-orange (salmon) color (Fig. 4), and lacking secondary annular grooves and dermal scale pockets.

##### Species comparisons.

Regarding the species of the genus *Caecilia*, the absence of secondary annular grooves distinguishes *C. pulchraserrana* sp. nov. from *C. abitaguae* Dunn, 1942, *C. albiventris* Daudin, 1803, *C. armata* Dunn, 1942, *C. antioquiaensis* Taylor, 1968, *C. bokermanni* Taylor, 1968, *C. dunni* Hershkovitz, 1938, *C. flavopunctata* Roze & Solano, 1963, *C. gracilis* Shaw, 1802, *C. guntheri* Dunn, 1942, *C. isthmica* Cope, 1878, *C. leucocephala* Taylor, 1968, *C. marcusi* Wake, 1985, *C. mertensi* Taylor, 1973, *C. museugoeldi* Maciel & Hoogmoed, 2018, *C. nigricans* Boulenger, 1902, *C. occidentalis* Taylor, 1968, *C. pressula* Taylor, 1968, *C. perdita* Taylor, 1968, *C. subnigricans* Dunn, 1942, *C. subterminalis* Taylor, 1968, *C. tentaculata* Linnaeus, 1758, *C. tenuissima* (Taylor, 1973), *C. thompsoni* Boulenger, 1902, and *C. volcani* Taylor, 1969.

*Caecilia pulchraserrana* sp. nov. shares with *C. attenuata* Taylor, 1968, *C. caribea* Dunn, 1942, *C. corpulenta* Taylor, 1968, *C. crassisquama* Taylor, 1968, *C. degenerata* Dunn, 1942, *C. inca* Taylor, 1973, *C. orientalis* Taylor, 1968, *C. pachynema* Günther, 1859, and *C. subdermalis* Taylor, 1968 the absence of secondary annular grooves and the presence of incomplete primary annular grooves. However, the new species can be distinguished from these nine species by having a lower number of primary annular grooves (100–104 vs. 114–199). *Caecilia pulchraserrana* sp. nov. most closely resembles *C. degenerata*, which also lacks subdermal scales, but differs from it in having fewer primary annuli.

##### Description of holotype.

An adult female (Fig. 3). Head dorsoventrally flattened and slightly narrower than body; head width at CM 63% of width at midbody, head width at CM 72% of head length; head length 3.5% of total length; interorbital distance 40% of head width. Snout projects 1.6 mm beyond mouth; tip of snout rounded in dorsal and lateral view (Fig. 3); area between the eye and naris flattened. Eyes visible but small, eye diameter 4% of head length and 13.5% of eye-nostril distance; nares small, margins slightly protuberant, directed posterodorsally, visible from above. Tentacular openings circular and small, slightly raised above skin, laterally positioned near margin of mouth (Type D sensu Lynch 1999, Fig. 3D, E), slightly closer to corner of mouth than to nostrils. Tongue anteriorly attached, surface smooth with some longitudinally oriented grooves. Teeth pointed, recurved, with size decreasing posteriorly; premaxillary-maxillary and dentary teeth monocuspid and visible externally. Premaxillary-maxillary teeth 13, posterior maxillary teeth smaller. Premaxillary-maxillary series extending behind level of choanae. Vomeropalatine teeth 10, monocuspid, relatively uniform, moderately recurved, not visible externally, similar in size. Dentary teeth 12, moderately recurved, faintly larger than premaxillary-maxillary teeth. Choanae subovoid; narial plugs visible (Fig. 3F). Nuchal grooves indistinct dorsally and ventrally, incompletely encircling body with transverse grooves on the collars, in ventral surfaces. First collar shorter than second. Body subcylindrical, slightly deeper than wide (Fig. 3A, B); body width at midbody 4% of total length. Width along body varies slightly, narrower at terminal region. Primary annuli 104 incomplete dorsally and ventrally. Primary annular grooves completely encircling the body. Secondary grooves absent (Fig. 3G–I). Dermal scale pockets absent. Vent circular; disc around vent conspicuous enlarged (Fig. 3I) with seven denticulations anterior, seven nearly equal posterior denticulatios (Fig. 3I); anal papillae absent, and unsegmented terminal shield of 4.9 mm length.

##### Color in life

(Fig. 4): Jaw margins, area between the eye and naris, and tentacular regions pink-orange (salmon); eyeballs completely violet blue (Fig. 4b); periorbital region salmon; body dark brownish with thin salmon-colored chromatophores; ventral surface of body slightly paler than dorsum; annular grooves on sides of body slightly darker than general body color.

##### Color in preservative

(ethanol 70%; Fig. 3): Body dark slate gray dorsally with diffuse khaki chromatophores; jaw margins, rostral and periocular regions yellowish; ventral and lateral surfaces slightly paler than dorsum; vent disk jaw margins and area between the eye and naris yellowish.

##### Variation of type series

(Tables 3, 4). There is little variation among type specimens. Head flattened and slightly narrower than body, head width at CM 58–97% of width at midbody; head width at CM 72–92% of head length; head length 2–4% of total length; interorbital distance 36–50% of head width. Eye diameter 4–8% of the head length and 10–19% of eye-nostril distance. Nares small, slightly protuberant, directed posterodorsally, and visible from above. Premaxillary-maxillary teeth 11–13. Vomeropalatine teeth 9–12. Dentary teeth 10–13. First collar 66–96% of second collar. Body width at midbody 2–4% of total length. Primary annuli incomplete dorsally and ventrally. Secondary grooves and dermal scales absent. Vent circular; disc around with 12–15 anal denticulations. Denticulations usually seven-eight anteriorly, and seven posteriorly, nearly equal in size (Fig. 3I).

**Table 2.** Morphological data of the Colombian species of *Caecilia* that lack secondary annular grooves and possess incomplete primary annular grooves. Abbreviations are given in Material and methods.

Species	PAG	TL (mm)	TL/D	Dermal scale pockets	Sample size	Source
*C. caribea*	142–152	390–585	53–55	Absent	4	Dunn 1942, Lynch 1999
*C. corpulenta*	129–132	152–441	19–35	Absent	6	Taylor 1968, Lynch 1999
*C. degenerata*	123–137	390–1050	38–58	Absent	9	Lynch 1999
*C. orientalis*	114–124	231–673	29–55	Present	8	Lynch 1999
*C. subdermalis*	116–138	131–680	28–54	Present	32	Lynch 1999
*C. pulchraserrana* sp. nov.	100–104	195–232	9–12	Absent	7	This study

**Table 3.** Morphometric (in mm) and meristic data of the type series of *Caecilia pulchraserrana* sp. nov. Abbreviations are given in Materials and methods.

	IAvH-Am-15487 Holotype	IAvH-Am-15490 Paratype	IAvH-Am-15489 Paratype	IAvH-Am-15488 Paratype	UIS-MHN-A-6575 Paratype
Sex	F	M	M	F	F
PAG	104	100	101	103	100
TL	206	214	200	232	195
HW	5.4	5.3	5.0	4.8	4.3
HWNG1	5.2	4.2	4.4	4.9	4.3
WC2	5.8	4.6	4.0	5.2	4.8
WMB	8.5	6.2	5.5	8.1	6.2
CMB	22	18	17	23	18
WBV	5.2	3.7	4.0	4.4	3.5
HL	7.4	5.8	6.4	6.0	5.1
HH	5.1	4.8	4.0	4.4	3.8
IND	1.7	1.6	1.5	2.0	1.2
IOD	2.9	2.6	2.3	2.8	2.5
ED	0.3	0.4	0.4	0.2	0.4
ND	0.18	0.18	0.16	0.16	0.15
END	2.3	2.3	1.6	2.5	2.1
STD	6.9	5.6	5.7	6.0	5.2
STND	0.8	0.6	0.4	0.7	0.7
STLPD	2.2	2.1	2.1	2.0	1.8
STOD	3.3	2.7	2.5	3.4	2.9
TA	0.27	0.19	0.30	0.26	0.33
INTA	2.3	2.2	1.8	2.3	1.9
TAOD	2.5	2.1	1.9	2.6	2.1
TALPD	1.0	1.3	0.6	1.4	0.99
TANRD	0.99	0.67	0.69	0.75	0.7
TASTD	0.6	0.7	0.7	0.2	0.7
LPOD	1.0	1.2	0.9	1.0	0.7
WCH	0.16	0.11	0.09	0.11	0.14
C1	1.6	1.2	1.6	1.1	0.9
C2	1.7	1.5	2.4	1.5	1.1
BH	7.0	4.4	4.1	6.5	5.1
ADD	2.9	2.6	2.9	2.7	2.6
VP	11	9	10	9	11
Premaxillary-maxillary teeth	13	11	14	14	12
Dentary teeth	12	13	10	11	12

**Table 4.** Ratios and percentages of measurements of the type series of *Caecilia pulchraserrana* sp. nov. Abbreviations are given in Materials and methods.

	IAvH-Am-15490 Paratype	IAvH-Am-15489 Paratype	IAvH-Am-15488 Paratype	IAvH-Am-15487 Holotype	UIS-MHN-A-6575 Paratype
Sex	M	M	F	F	F
C1/C2	75.9	66.1	70.5	96.4	82.7
TL/D	11.8	11.7	10.0	9.3	10.8
TL/HL	39.9	40.0	48.2	38.1	44.5
TL/ WMB	34.1	35.9	28.3	24.1	31.5
L/HW	36.7	30.8	38.2	27.7	37.8
HL/HW	92.0	77.0	79.2	72.9	85.0

##### Distribution and natural history.

**Figure 6.**
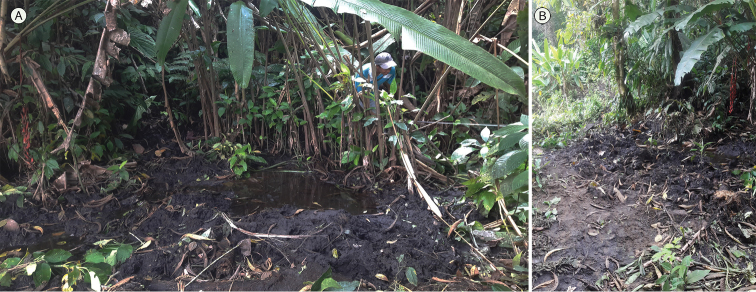
Habitat of *Caecilia pulchraserrana* sp. nov. in the Serranía de los Yariguíes in Santander Department, El Carmen de Chucurí Municipality, vereda La Belleza, Cascajales River, 06°34'8.9"N, 73°34'20.2"W, 789 m a.s.l.. **A** View showing standing water in marshy area **B** Transitional change of wetter (right) to drier (left) microhabitat.

*Caecilia pulchraserrana* sp. nov. is currently known from two adjacent, relictual tropical wet forest localities on the western slope of the Cordillera Oriental of Colombia (Serranía de los Yariquíes; Fig. 1) at elevations between 731–789 m a.s.l. The Serrania of the Yariguies corresponds to an isolated mountain range that is part of the western slope of the Cordillera Oriental of Colombia (Fig. 1). *Caecilia pulchraserrana* sp. nov. is a fossorial species associated with marshy areas surrounded by secondary vegetation at the forest edge (Fig. 6). The specimens were collected during the dry season in very wet soils lacking rocks (i.e., bogs; Fig. 6), in a slightly inclined area (nearly 5°of slope) covered with vegetation of the family Heliconiaceae (*Heliconia* spp., Fig. 6).

*Caecilia pulchraserrana* sp. nov. was obtained during the initial 10 minutes of removal with a hoe.We extracted the first specimen in intermediate substrates between marshy and dry areas; after 40 minutes of excavation in these selected areas, we obtained four additional specimens. Using these same criteria, when moving two kilometers above the original point, an area with similar characteristics was located and within 20 minutes we collected two additional specimens. *Caecilia pulchraserrana* sp. nov. was collected on black sandy soils with high organic matter content. These caecilians move quickly under the substrate, so once the first specimen is detected it is important to quickly create channels to surround and block them from escaping.

##### Etymology.

The specific epithet is formed from the Latin *pulchra* (nominative feminine singular of *pulcher*), meaning beauty, and the Spanish adjective *serrana* (feminine singular of *serrano*), from the sierra or serranía. This specific name refers to the type locality of the species: vereda La Belleza (beauty in English) in the western foothills of the Serranía de Los Yariguíes. The specific name was chosen using a citizen science approach. First, scientists and inhabitants of the El Carmen de Chucurí municipality gathered a list of possible names for the new species. Then, the list of potential names and their meanings was shared with the local people, who voted to choose their preferred name.

## Discussion

### Phylogenetic relationships

Our description of *Caecilia pulchraserrana* sp. nov. brings the number of known species of *Caecilia* to 35 (Frost 2018). Molecular data are currently available for only six of these species (including the three newly sequenced species analyzed here), which precludes a thorough analysis of the relationships within the genus. Consequently, our phylogenetic analysis (Fig. 2) was designed mainly to test the generic placement of *C. pulchraserrana* sp. nov. in addition to exploring the relationships of *C. isthmica* and *C. thompsoni* (two other species that are endemic to Colombia). Our results recovered *C. pulchraserrana* sp. nov., *C. isthmica*, and *C. thompsoni* within *Caecilia* (Fig. 2), corroborating the generic placement of the new species and the monophyly of the genus, as previously hypothesized by Wilkinson et al. (2011). Our analysis recovered *Oscaecilia* as the sister group of *Caecilia*, which agrees with the results of San Mauro et al. (2014) but disagrees with those of Pyron and Wiens (2011), who instead recovered *Caecilia* as paraphyletic with respect to *Oscaecilia*.

Our phylogenetic analysis only included two mitochondrial loci and a small number of species and should not be considered as a robust resolution of caecilian relationships. Nevertheless, our results highlight several potential cases of non-monophyletic taxa and suggest that a taxonomic revision, including a major generic rearrangement, is warranted. Our study includes, for the first time, the Colombian endemics Epicrionops aff. parkeri (Rhinatrematidae) and *Microcaecilia nicefori* in molecular phylogenetic analyses. On one hand, recent contributions (Maciel et al. 2018) have allowed taxonomic rearrangements within Rhinatrematidae, with *Rhinatrema nigrum* and *R. bivittatum* being recovered as monophyletic, supporting previous claims (Wilkinson and Gower 2010; Wilkinson et al. 2011; Pyron and Wiens 2011; San Mauro et al. 2014) that *Epicrionops* could be transferred to *Rhinatrema*. Our analysis recovers Epicrionops aff. parkeri nested within a monophyletic *Epicrionops* (*E. marmoratus*+ E. aff. parkeri with 89%), which was sister to *Rhinatrema* (Fig. 2), corroborating the results obtained by Maciel et al. (2018).

On the other hand, *Microcaecilia nicefori* was recovered as the sister taxon to a clade formed by the dermophiids *Gymnophis multiplicata* + *Dermophis mexicanus* and the remaining siphonopids, including *Microcaecilia*, *Brasilotyphlus guarantanus*, *Siphonops*, and *Luetkenotyphlus*. In addition, *Microcaecilia* and *Siphonops* were recovered as paraphyletic with respect to *Brasilotyphlus guarantanus* and *Luetkenotyphlus brasiliensis*, respectively (Fig. 2). Recently, Correia et al. (2018) also presented evidence that *Microcaecilia* is paraphyletic with respect to *Brasilotyphlus*. The placement of *Luetkenotyphlus brasiliensis* within *Siphonops* contrasts with results of Pyron and Wiens’ (2011) and Maciel et al.'s (2019) analyses that found *Luetkenotyphlus* and *Siphonops* to be sister taxa. Although analyses by San Mauro et al. (2006), San Mauro et al. (2014) and Correia et al. (2018) also recovered *Luetkenotyphlus* and *Siphonops* as sister groups, these studies only included one species of *Siphonops* (*S. annulatus*). Therefore, additional molecular data are needed to clarify the delimitation of these clades.

Consistent with previous findings (i.e., Correia et al. 2018), our phylogenetic analysis recovers *Microcaecilia* as non-monophyletic. Previously, based on evidence from dentition (relationship between VPs and rows of PM) and orbit (open versus closed orbit), Wilkinson et al. (2013) suggested that some *Microcaecilia*, including the type species of the genus (*Dermophis albiceps* Boulenger, 1882; not included herein), are more closely related to *M. nicefori* (*Gymnophis nicefori* Barbour, 1925, the type species of *Parvicaecilia*, currently in the synonymy of *Microcaecilia*; analyzed here for the first time) than to other species of *Microcaecilia*. That is, the position of trans-Andean *Microcaecilia nicefori* compared to other cis-Andean members of the genus suggests the revalidation of the genus *Parvicaecilia*. However, our analysis does not represent solid evidence due to several aspects, such as the low number of genes used, the low support values (a bootstrap value of only 45%), and the absence of key terminals, such as the type species of the Amazonian *Microcaecilia* (*M. albiceps* (Boulenger, 1882). Thus, inclusion of relevant taxa, such as *M. albiceps*, in future phylogenetic analyses is key to guiding taxonomic changes. At the interfamilial level, our results provide evidence for the first time that Shiphonopidae is paraphyletic with respect to Dermophiidae due to the placement of *M. nicefori* (Fig. 2). Additional, large scale phylogenetic studies are required to rigorously test this finding.

### Status of the trans-Andean populations of *Caecilia degenerata*

Lynch (1999) suggested that *Caecilia degenerata* is restricted to the Cordillera Oriental of Colombia (Departments of Boyacá, Cundinamarca and Santander). However, morphological and biogeographical evidence suggests that the cis- and trans-Andean populations are not conspecific. The type series was collected at two cis-Andean localities: Garagoa (Boyacá Department), the type locality, and Choachí (Cundinamarca Department), ca. 90 km southwest of the type locality (Dunn, 1942). Later, Ruiz-Carranza et al. (1996) and Lynch (1999) examined a series of trans-Andean specimens collected at Muzo (Boyacá Department), Tena and Sasaima (Cundinamarca Department), and Charalá (Santander Department), and referred them to *C. degenerata*, based on morphological similarity and (presumably) relative geographical proximity. Although the absence of secondary annular grooves, the number of primary annular grooves (127–138 in the cis-Andean populations vs. 123–137 in the trans-Andean populations), and the ratio of length/diameter (32–60 in the cis-Andean populations vs 48–58 in the trans-Andean populations; Ruiz-Carranza et al. 1996, Lynch 1999) are consistent with the hypothesis of conspecific populations. The cis- and trans-Andean populations are isolated by biogeographic barriers that includes high and steep mountains, xerophytic areas, and rainy environments, factors that usually play a fundamental role in the speciation of Andean amphibians (Lynch et al. 1997). To test the conspecificity of the populations of *C. degenerata*, a more extensive sampling of specimens, populations, and additional molecular data are required. Finally, although Taylor (1968) recorded specimens of *C. degenerata* in Tomaque (probably in Colombia or Peru) and Río Pache (probably in Peru), we agree with Lynch (1999) that *C. degenerata* is restricted to the (eastern) Cordillera Oriental of Colombia.

## Conclusions

*Caecilia pulchraserrana* sp. nov. is described as an endemic species from the Serranía de los Yariguies. The species is similar to *C. degenerata*, from which it can be distinguished using morphological characters. According to their morphology, we hypothesize there is a group of closely related species that comprises *C. caribea*, *C. corpulenta*, *C. degenerata*, *C. orientalis*, and *C. subdermalis*. The trans-Andean Microcaecilia nicefori is an endemic and poorly known species from Colombia. We provide here the first analysis of molecular data that tests its phylogenetic position. Our results address the need to evaluate with more evidence the status of the genus *Parvicaecilia* Taylor, 1968 (currently under the synonymy of *Microcaecilia*), and the potential non-monophyly of the family Siphonopidae. Further analyses sampling additional taxa and molecular markers are required to establish a more robust classification for Gymnophiona.

## Supplementary Material

XML Treatment for Caecilia pulchraserrana

## Appendix 1

Additional specimens examined in this study. Number of specimens examined of each species in parenthesis.

***Caecilia guntheri*** (2): COLOMBIA: NARIÑO: La Planada Natural Reserve, 7 km South of Chucunes, 1780 m above sea level; IAvH-Am-1396; RISARALDA: Pueblo Rico Municipality, Vereda Montebello, Montezuma Reserve, 4°33'40.5"N, 74°21'4.9"W, 1650 m above sea level, IAvH-Am-8872.

***Caecilia isthmica*** (1): COLOMBIA: SUCRE: San Benito Abad Municipality, Vereda La Caimanera, site La Caimanera, 9°2'33.7"N, 74°54'17.6"W, 26 m above sea level, IAvH-Am-8246 (tissue IAvH-CT-22982).

***Caecilia subdermalis*** (10): COLOMBIA: CALDAS: Norcasia Municipality, Hidromiel camp, 5°34'16.4"N, 74°53'24.8"W, 850 m above sea level. IAvH-Am-9663; HUILA, Acevedo Municipality, Cueva de los Guácharos National Natural Park, 1820 m above sea level. IAvH-Am-0687, IAvH-Am-3541, IAvH-Am-3549, IAvH-Am-4316-7, IAvH-Am-4322-23, IAvH-Am-4708, IAvH-Am-5388.

***Caecilia thompsoni*** (1): COLOMBIA: CUNDINAMARCA: La Mesa Municipality, site Payacal, La Gran Via, Tacarcuna Farm, 04°39'6,77"N, 74°25'1.0"W; 1100 m above sea level, MUJ 3713 (tissue IAvH-CT-22986).

**Epicrionops aff. parkeri** (2): COLOMBIA: ANTIOQUIA: municipality of El Carmen de Viboral, vereda El Porvenir, creek afferent to the Melcocho River, 5°54'7.9"N, 75°10'25.6"W, 898 m above sea level, IAvH-Am-14608, IAvH-Am-14609 (tissue IAvH-CT-21477).

***Microcaecilia nicefori*** (1): COLOMBIA: TOLIMA: municipality of Coello, El Neme farm (outside of town), 4°7'12.50"N, 74°55'21.10"W, 327 m above sea level, IAvH-Am-14879 (tissue IAvH-CT-22985).

***Typhlonectes natans*** (2): COLOMBIA: SUCRE: San Benito Abad Municipality, Vereda La Caimanera, site La Caimanera, 9°27'1"N, 74°54'26.7"W, 25 m above sea level, IAvH-Am-8275 (tissue IAvH-CT-22983). NORTE DE SANTANDER: San José de Cúcuta Municipality, Aguasal Creek, Footbridge about 1.2 km northeast of the community of Aguasal, 08°13'05"N, 072°32'31.2"W, 62 m above sea level, IAvH-Am-14559 (tissue IAvH-CT-22984).
